# Modulation of Adult Hippocampal Neurogenesis by Sleep: Impact on Mental Health

**DOI:** 10.3389/fncir.2017.00074

**Published:** 2017-10-12

**Authors:** Cristina Navarro-Sanchis, Olivier Brock, Raphaelle Winsky-Sommerer, Sandrine Thuret

**Affiliations:** ^1^Department of Basic and Clinical Neuroscience, Institute of Psychiatry, Psychology and Neuroscience, King's College London, London, United Kingdom; ^2^Surrey Sleep Research Centre, Department of Clinical and Experimental Medicine, Faculty of Health and Medical Science, University of Surrey, Guildford, United Kingdom

**Keywords:** neurogenic niche, plasticity, cognition, sleep disruption, memory, psychiatric disorders, mood, depression

## Abstract

The process of neurogenesis has been demonstrated to occur throughout life in the subgranular zone (SGZ) of the hippocampal dentate gyrus of several mammals, including humans. The basal rate of adult hippocampal neurogenesis can be altered by lifestyle and environmental factors. In this perspective review, the evidence for sleep as a modulator of adult hippocampal neurogenesis is first summarized. Following this, the impacts of sleep and sleep disturbances on hippocampal-dependent functions, including learning and memory, and depression are critically evaluated. Finally, we postulate that the effects of sleep on hippocampal-dependent functions may possibly be mediated by a change in adult hippocampal neurogenesis. This could provide a route to new treatments for cognitive impairments and psychiatric disorders.

## Introduction

Neurogenesis is the process by which new neurons arise from neural stem and progenitor cells, mature, specialize and become integrated and functional within the neuronal network. Although originally believed to occur solely during the early stages of development, it was later demonstrated to be present in adult birds (Goldman and Nottebohm, 1983), rodents (Altman and Das, 1965; van Praag et al., 1999), monkeys (Kornack and Rakic, 1999), and even humans (Eriksson et al., 1998; Spalding et al., 2013). Neurogenic niches have been recorded in various specific areas of the central nervous system in adult rodents, namely the subventricular zone (SVZ) lining the lateral ventricles, where neuroblasts originate and newborn neurons eventually migrate to the olfactory bulb (Altman, 1969), and the subgranular zone (SGZ) of the hippocampal dentate gyrus where new neurons migrate a short distance through the granule cell layer (Altman and Das, 1965). In adult humans, evidence supports the existence of a similar neurogenic niche in the hippocampal SGZ (Spalding et al., 2013) but neuroblasts originating from the human SVZ appear to migrate into the striatum (Ernst et al., 2014).

In this review, we focus on the hippocampal neurogenic niche. The hippocampus has many interesting higher level functions which these newly formed neurons could potentially become part of, such as mood and emotion (Miller and Hen, 2015) and learning and memory (Aimone et al., 2014). Therefore, a change in neurogenesis could affect these hippocampal-dependent functions. For example, there is experimental evidence linking decreased neurogenesis to impaired learning, especially in the spatial domain (Shors et al., 2001; Thuret et al., 2009), decreased memory retention (Deng et al., 2010), and pattern separation (Clelland et al., 2009). On the other hand, hippocampal-dependent learning tasks appear to increase neurogenesis (Dobrossy et al., 2003). Likewise, computational modeling of the effects of adult neurogenesis on hippocampal function has generated different theories for the role of newborn neurons. These include encoding of temporal information into memories, avoidance of memory interference and cognitive flexibility during learning of new tasks and balancing pattern separation/integration (reviewed in Gonçalves et al., 2016). A reduction in neurogenesis has also been associated with depressive symptoms (Egeland et al., 2017), and antidepressant treatments with an increase in neurogenesis (Grassi Zucconi et al., 2006; Sahay and Hen, 2007; Anacker et al., 2011). Overall, new theories are emerging linking regulation of dentate gyrus functions by adult neurogenesis affecting the cognitive processes which have important implications for both memory and mood (Anacker and Hen, 2017).

Neurogenesis in adults does not occur at a constant rate and is affected by many endogenous and exogenous factors, including hormones (Tanapat et al., 1999), physical and psychosocial stress (Gould et al., 1999), physical activity (Brown et al., 2003), diet (Murphy et al., 2014), enriched environments (Brown et al., 2003), and age (Kuhn et al., 1996). Sleep- and circadian rhythms have also been suggested to be key modulators of plasticity in neural networks (Abel et al., 2013; Frank and Cantera, 2014), resulting in alterations in learning and memory, cognitive performance, as well as emotion regulation (Lo et al., 2012; Krause et al., 2017). In this context, a role for sleep specifically in adult neurogenesis has been investigated in the past decades (Guzman-Marin et al., 2003; Hairston et al., 2005).

This review will summarize and critically evaluate the evidence of the role of sleep as a modulator of adult hippocampal neurogenesis, which primarily emerges from studies of acute and chronic sleep disruption in rodents. We will then discuss the effects of sleep disruption on hippocampal-dependent cognitive functions in rodents and humans. Lastly, we will hypothesize that the negative effects of sleep disturbances on hippocampal-dependent cognitive functions in humans may possibly occur due to a decrease in adult hippocampal neurogenesis. If this hypothesis is ascertained, it could open new avenues for research and treatments of cognitive disorders and psychiatric diseases.

## Modulation of adult hippocampal neurogenesis by sleep in rodents

### Sleep and the hippocampus

Sleep is divided into two types, i.e., rapid-eye-movement (REM) sleep, also known as paradoxical sleep, and non-REM (NREM) sleep, which encompass deep slow wave sleep. NREM and REM sleep can be measured and identified using electroencephalogram (EEG) recordings (Dijk, 2009). REM and NREM sleep are characterized by specific oscillatory rhythms in the EEG, such as theta rhythms in REM sleep, while slow waves, spindles and sharp wave ripples are observed during NREM sleep. Some of these EEG rhythms are associated with the hippocampus, such as theta oscillations in REM sleep and high frequency sharp wave ripples in NREM sleep which involve 10 to 18% of hippocampal neurons (Watson and Buzsaki, 2015). A close temporal association between hippocampal sharp wave ripples and sleep spindle oscillations arising from the thalamo-cortical networks has been established (Siapas and Wilson, 1998; Sirota et al., 2003). Furthermore, a temporal coupling has also been observed with cortical slow oscillations (Sirota et al., 2003; Molle et al., 2006; Sullivan et al., 2011). These EEG rhythms have been proposed to contribute to memory consolidation (Buzsaki et al., 1987; Buzsaki, 1989; Wilson and McNaughton, 1994; Girardeau et al., 2009; Karlsson and Frank, 2009; Buzsaki and Silva, 2012), transmitting information from the hippocampus to the cortex and other regions (Buzsaki, 1989; Sirota and Buzsaki, 2005; Hahn et al., 2007). The different sub-regions of the hippocampus (i.e., CA1, CA3, DG) contribute to memory consolidation, and are characterized by specific population activity patterns that can be temporally coupled (Daumas et al., 2005; Abel et al., 2013; Aton et al., 2014; Josselyn et al., 2015). In the DG, dentate spikes are triggered by the entorhinal cortex and associated with hilar interneuron activity, while they suppress the CA3-CA1 population activity during SWS (Bragin et al., 1995). A recent study identified that CA1 parvalbumin-expressing interneurons show enhanced firing coherence with CA1 theta activity and mediate hippocampal network oscillations involved in memory consolidation (Ognjanovski et al., 2017).

### Chronic sleep disruption

Many studies over the past decades have investigated a potential role of sleep on neurogenesis, primarily by using chronic sleep disruption over a large range of durations in rodents (Guzman-Marin et al., 2003, 2005, 2007; Hairston et al., 2005; Roman et al., 2005; Tung et al., 2005; Mirescu et al., 2006; Sportiche et al., 2010; Mueller et al., 2015), as summarized in Table 1. Sleep disruption have included total sleep deprivation, sleep restriction, sleep fragmentation, as well as relatively selective REM sleep deprivation. Thus, methods to induce deprivation/disruption of sleep are numerous, including treadmill running (Hairston et al., 2005; Roman et al., 2005; Mirescu et al., 2006; Sportiche et al., 2010), slow rotating wheels (Guzman-Marin et al., 2005), and over the water platforms (Guzman-Marin et al., 2003; Tung et al., 2005). These methodologies are all associated with potential confounding effects (e.g., stress, imposed locomotion) but appropriate controls can address some of these confounders (e.g., locomotion). Another aspect that may also contribute to discrepancies between studies is the time of day at which experiments are performed, as many physiological mechanisms display daily circadian rhythms. Studies addressing this caveat have not led to a consensus on whether aspects of neurogenesis exhibit daily rhythms (reviewed in Meerlo et al., 2009; Fernandes et al., 2015; Mueller et al., 2015), requiring thus further experiments. Despite this, some coherent results have been obtained across methodologies, suggesting that sleep disturbances *per se* might be responsible for the changes in neurogenesis.

**Table 1:** Studies investigating chronic sleep disturbance and adult hippocampal neurogenesis.

**Reference**	**Animal model**	**Sleep disturbance paradigm**	**Adult hippocampal neurogenesis readout**
Guzman-Marin et al., 2003	Adult male Sprague-Dawley rats	SD for 96 h Treadmill vs. YC vs. CC	Cell proliferation in DG–54 % reduction in SD vs. YC (*p* < 0.001) and 68 % in SD vs. CC (*p* < 0.001)
Tung et al., 2005	Adult male Sprague-Dawley rats	SD for 56 h vs. 48 h + 8 h recovery sleep Disc over water vs. CC	Cell proliferation in DG–36% reduction in 56 h SD and 39% in 48 h SD + 8 h recovery sleep vs. CC Suppression twice as large in posterior as anterior hippocampus.
Mirescu et al., 2006	Adult male Sprague-Dawley rats	SD for 72 h Platform vs. YC vs. CC for 72 h	Cell proliferation in GCL–reduced in SD, *p* = 0.018 Cell survival (1 week) in GCL–reduced in SD (*p* = 0.006) Cell survival (3 weeks) in GCL–reduced in SD (*p* = 0.038) Cell proliferation in SVZ–no difference (*p* = 0.14) Cell differentiation of newborn cells in GCL–70% (in)mature neurons (1 week, TuJ1), 80% mature neurons (3 weeks, NeuN)
Guzman-Marin et al., 2005	Adult male Sprague-Dawley rats	SD for 96 h Intermittent treadmill vs. YC	Cell survival (3 weeks) in DG–39.6% reduction in SD Cell differentiation into mature neuron (NeuN)–25.3% reduction in SD (*p* < 0.001) Cell differentiation into immature neuron (DCX) and gliogenesis (S100 betta)–no difference (*p* > 0.05)
Hairston et al., 2005	Adult rats	SR for 6 h Enriched environment vs. control Trained on spatial vs. non-spatial task	Cell survival (17 days) in DG–reduction in SR (*p* = 0.033) Cell differentiation into immature neurons (DCX)–increased in non-SR spatial (*p* < 0.05) Performance in spatial task–decreased in SR (*p* = 0.050) Performance in non-spatial task–enhanced in SR (*p* = 0.045)
Sportiche et al., 2010	Adult male Sprague-Dawley rats	SF for 12 days Intermittent treadmill vs. SF controls (SFC) vs. treadmill controls (TC) vs. CC Barnes maze 2 weeks post SF, 5 days same escape, then 2 days rotated position	Cell survival (30 days) in DG–32% reduction in SF vs. SFC and TC (*p* < 0.05) Cell differentiation into mature neurons (NeuN)–no difference in SF vs. SFC (*p* > 0.4) Performance (progressive decrease in escape time)–decreased in SF vs. SFC (*p* = 0.08) and vs. TC and CC (*p* < 0.05) Random attempts–increased in SF (*p* < 0.001)
Roman et al., 2005	Adult male Wister rats	SR (4 h undisturbed sleep/day) for 8 days Slowly rotating wheel vs. YC vs. CC	Cell proliferation–reduction in SR in hilus (*p* = 0.039) and in SR and YC in SGZ (*p* = 0.002) Cell differentiation into mature neurons (NeuN) and gliogenesis (GFAP)–no difference (*p* = 0.94)
Mueller et al., 2008	Adult male Long Evans rats	SD for 96 h Platform vs. YC vs. CC	Cell proliferation in DG–46% reduction in SD vs. YC and 52% reduction vs. CC (*p* = 0.0001) Cell differentiation into immature neurons (DCX)–no effect (*p* = 0.94)
Guzman-Marin et al., 2007	Adult male Sprague-Dawley rats	SF for 4 and 7 days Intermittent treadmill vs. YC	Cell proliferation in DG–70% reduction in 4 days SF (*p* < 0.01) and in 7 days SF (*p* < 0.001) Cell differentiation (3 weeks) into mature neurons (NeuN)–52% reduction in 4 days SF; 22% reduction in 7 days SF (*p* < 0.05) Gliogenesis (S100 beta)–no difference

*SD, sleep deprivation; SR, sleep restriction; SF, sleep fragmentation; YC, yoked control; CC, cage control; DG, dentate gyrus; GCL, granule cell layer; SVZ, subventricular zone*.

As neurogenesis depends on cell proliferation and cell survival, maturation and differentiation, it is important to categorize and scrutinize the readouts of these reports separately to better understand the impact of sleep on the neurogenic process. Cell proliferation and survival are generally quantified using bromodeoxyuridine (BrdU), while the identification of neurons is achieved using in addition a neural marker such as NeuN (Mullen et al., 1992). Hence, cells co-expressing these factors are identified as newly formed neurons. Studies evaluating sleep disruption on adult neurogenesis have primarily focused on cell proliferation, survival and differentiation, while the integration of newborn neurons in existing neural circuits has received less attention.

#### Total sleep deprivation and effects on cell proliferation

Total sleep deprivation over a period of 48 h or more has consistently been reported to decrease the basal rate of cell proliferation in the dentate gyrus from 30 to 80% (Table 1). Guzman-Marin and colleagues (Guzman-Marin et al., 2003) sleep-deprived adult Sprague-Dawley rats for 96 h by subjecting them to forced locomotion in a treadmill. Rats exhibited a 54% reduction in proliferation in the dorsal hippocampus when compared to treadmill controls (i.e., displaying similar amount of locomotor activity) and a 68% reduction when compared to home-cage controls. However, rats were euthanized 48 h after BrdU administration, which allowed time for further mitotic cycles and/or apoptosis. A similar experiment by Tung and colleagues (Tung et al., 2005) used a disc-over-water paradigm to induce REM sleep deprivation for 56 h. BrdU was injected 2 h before tissue collection, thus ensuring only cells in the S phase were labeled. The study showed a 36% decrease in cell proliferation, confirming the effect across sleep disruption methodologies. In addition, this reduction lasted after 8 h of sleep recovery following deprivation, showing that the effects of sleep deprivation on neurogenesis were not normalized within this 8 h sleep recovery period. This specific decrease was mostly observed in the posterior dentate gyrus. Later studies have confirmed the specificity of sleep deprivation to dentate gyrus neurogenesis, while neurogenesis in the SVZ was not altered (Mirescu et al., 2006).

#### Total sleep deprivation and effects on cell survival, maturation and differentiation

Other studies investigated the effects of sleep deprivation on cell survival and cell differentiation. Guzman-Marin et al. (2005) sleep-deprived rats for 96 h using an intermittent treadmill and administered BrdU 3 weeks before neurogenesis assessment (Table 1). They observed a 39.6% decrease in BrdU-positive cells in sleep-deprived rats compared to treadmill controls, and significant differences in the percentage of cells co-labeling BrdU and NeuN (46.6 vs. 71.9% respectively) (Table 1).

Other studies investigating cell survival, maturation and differentiation used other sleep disruption approaches, such as sleep fragmentation and sleep restriction are discussed below.

#### Sleep fragmentation and sleep restriction

While total sleep deprivation is a well-established and frequently used methodology to assess the contribution of sleep to physiological processes, other types of disruptions, such as sleep fragmentation or sleep restriction, are closer to sleep disturbances in humans comorbid with many psychiatric disorders (Baglioni et al., 2016). Hairston et al. (2005) reduced the amount of sleep by 50% for 4 days using an enriched environment while rats were being trained on spatial (hippocampal-dependent) and non-spatial (non-hippocampal dependent) tasks. A decrease in cell proliferation and cell survival was observed in sleep-restricted rats, as well as a decrease in neural differentiation in sleep-restricted rats which were trained on the spatial task. Sleep fragmentation induced by enforced locomotion, using an intermittent treadmill, for 4 or 7 days also induced a 70% reduction of BrdU- and Ki67-labeled cells (Ki67, marker of cell proliferation) (Guzman-Marin et al., 2007). The same study showed that the number of cells expressing a neuronal phenotype 3 weeks after BrdU injection also decreased by 52% and 22% respectively. Sportiche et al. (2010) also reported a 32% decrease in BrdU-labeled cells after 12 days of sleep fragmentation, while Guzman-Marin et al. (2007) examined cell differentiation 3 weeks after a 1-, 4-, and 7-day sleep fragmentation paradigm, and reported a 30, 52, and 22% reduction of cells expressing a neuronal phenotype respectively. Rats subjected to 7-day sleep fragmentation also showed less suppression of REM sleep (presumably due to an increased homeostatic drive for REM sleep) which could account for the reduced suppression of cell differentiation (Table 1).

#### REM sleep deprivation

Studies further investigated whether a specific sleep stage may be primarily associated with neurogenesis, using different methodologies to induce sleep disruptions. Several studies used the platform over water method (Mirescu et al., 2006; Mueller et al., 2008) and showed reduced cell proliferation. Of note, while this paradigm primarily suppresses REM sleep, it also decreases the amount of NREM sleep. Guzman-Marin et al. (2008) used an alternative approach to attempt to selectively disrupt REM sleep during 4 days, with a treadmill being triggered when rats enter REM sleep assessed by online EEG recordings. Cell proliferation was 63% lower in REM sleep-deprived (REMD) rats compared to yoked controls (YC) and 82% lower than observed in home-cage controls. Strikingly, all groups showed a positive correlation between cell proliferation and the percentage of REM sleep. These results suggest that lack of REM sleep plays a key role in reduced neurogenesis induced by sleep deprivation, irrespective of the methodology used and their potential confounding effects (e.g., stress). Although REMD and YC rats did not differ significantly regarding amount of NREM sleep or EEG-derived slow wave activity which quantifies the slow wave oscillations (1–4 Hz) characteristic of deep NREM sleep, it cannot be ruled out that the REMD procedure may induce other changes, including in waking behaviors, essential for the proliferative process. In contrast, a selective effect of REM sleep on cell maturation remains unclear, with some studies showing no significant differences (Table 1), while neuronal maturation was lower in REM sleep-deprived rats using a more selective REM sleep deprivation method (Guzman-Marin et al., 2008).

#### Potential mechanisms contributing to the effects of sleep disruption: stress, circadian rhythmicity or sleep disruption *per se*?

Even though there is a consensus on the negative effects of chronic sleep disturbances on neurogenesis, the mechanisms by which it occurs remain controversial. As acute sleep deprivation has not consistently been shown to decrease neurogenesis (see section below) and recovery is not immediate (Tung et al., 2005), the effects of sleep disruption may be indirect.

##### Stress and glucocorticoids

It has been established that stress affects neurogenesis (Mirescu and Gould, 2006) and sleep deprivation methods have been associated with an activation of the hypothalamo-pituitary-adrenal axis, widely assessed by increased levels of stress hormones such as corticosterone and adrenocorticotropic hormone (Tartar et al., 2009; Mongrain et al., 2010). Hence, the observed effects of sleep disruption on neurogenesis may be partly mediated by an increase in glucocorticoid levels and not a lack of sleep *per se*. Mirescu et al. (2006) reported that the reduction of neurogenesis observed in rats sleep deprived for 72 h was associated with elevated corticosterone levels. Importantly, using adrenalectomy combined with corticosterone supplementation in drinking water (to prevent corticosterone increase) abolished this reduction in cell proliferation. A further study by Guzman-Marin et al. (2007) reported that adrenalectomised rats with corticosterone replacement subjected to sleep fragmentation showed 55% fewer BrdU-positive cells when compared with adrenalectomised controls.

##### Circadian regulation

A plethora of functions in the hippocampus show circadian rhythmicity. For instance, a recent study demonstrated using fMRI scheduled throughout the 24 h cycle that the response of the hippocampus during a sustained attention task exhibited 24 h rhythmicity (Muto et al., 2016). Neural progenitor cells have a cell cycle of 24.7 h (Cameron and McKay, 2001), suggesting the possibility of circadian control. Hippocampal neurogenesis was suggested to be circadian-dependent under certain conditions (Goergen et al., 2002). Furthermore, a mouse study showed that exercise significantly increased neurogenesis only when administered at specific times during the day (Holmes et al., 2004). This could suggest that neurogenesis might be regulated by hippocampal clock genes. For instance, Per1, Cry, or Clock genes alter cell growth and cell cycle progression in mice (Matsuo et al., 2003; Gery et al., 2006; Miller et al., 2007). Corticosterone (CORT), brain-derived neuropathic factor (BDNF) (Sairanen et al., 2005; Rossi et al., 2006), and melatonin have also been proposed as mediators in the effect of circadian rhythms on hippocampal function (Ramirez-Rodriguez et al., 2011). At the molecular level, a circadian-controlled gene expression has been observed in the hippocampus in both rodents and humans (Jilg et al., 2010). In addition, sleep disruption methods alter the 24 h rest-activity rhythms and related waking behaviors, such as drinking and eating patterns, or nesting behavior. However, the contribution of circadian rhythms to different aspects of neurogenesis has been relatively unexplored. Circadian disruption imposed with a repeated “jet lag” protocol (i.e., twice-weekly phase advanced for 4 weeks) induced a reduction in cell proliferation and neurogenesis in hamsters, associated with impaired hippocampal-dependent learning and independent of glucocorticoids alterations (Gibson et al., 2010). Another study using chronic jet lag in Wistar rats suggests that the effects of circadian disruption are dependent on the type and duration of shifts, with phase advance (i.e., “*traveling eastbound*”) being associated with a greater decrease in the number of immature neurons compared to phase delay (“*traveling westbound*”) (Kott et al., 2012). Constant bright light conditions in rats, another approach to induce short- and long-term disruption of circadian rhythms did not affect cell proliferation and survival (Mueller et al., 2011). Moreover, several core circadian clock genes have also been implicated in adult hippocampal neurogenesis. *Bmal1* knockout mice, characterized by arrhythmic wheel-running activity and loss of circadian rhythmicity for clock gene expression, showed no significant difference in cell proliferation levels compared to wild-type controls (Rakai et al., 2014). However, the number of pyknotic cells, a proxy of cell death, was reduced and cell survival was enhanced, suggesting a possible role of the *Bmal1* clock gene and circadian rhythmicity in several aspects of neurogenesis. Another clock gene, *Period2*, was proposed to contribute to cell proliferation, generation of immature newborn neurons and cell survival in the dentate gyrus (Borgs et al., 2009). Interestingly, a study showed a diurnal rhythms of neurogenesis in the olfactory bulb in crustaceans, with the highest rate occurring at the most active time, i.e., dusk (Goergen et al., 2002). This raises the possibility that hormones and exercise influence neurogenesis within a circadian control pathway. Further investigation is needed to further disentangle the contribution of sleep *per se* from circadian rhythmicity in neurogenesis.

Other underlying mechanisms have been proposed to contribute to the effects of sleep disruption on neurogenesis, among which alterations in neurotransmitter systems and growth factors (Meerlo et al., 2009; Garcia-Garcia et al., 2011; Mueller et al., 2015).

### Acute sleep disruption

The effect of acute sleep deprivation on cell proliferation and neurogenesis remains elusive. An increase in cell proliferation following 12 h gentle handling in rats resulting in 80% wakefulness has been reported in the dentate gyrus, while no effects were observed in the SVZ (Grassi Zucconi et al., 2006). By contrast, Guzman-Marin et al. (2007) reported no difference in cell proliferation following 1-day of sleep fragmentation induced by automated intermittent treadmill activation, while the percentage of new cells expressing a neuronal phenotype 3 weeks after BrdU injection was reduced. One-day sleep restriction allowing 4 h of undisturbed sleep decreased cell proliferation in the hilus (Roman et al., 2005). Several studies reported no significant difference after 1 day of sleep deprivation using various paradigm, e.g., small platform (Mirescu et al., 2006), intermittent treadmill (Guzman-Marin et al., 2008) or deprivation by gentle procedures (van der Borght et al., 2006).

While several studies showing differences regarding species, age, strains, methods and/or durations led to consistent results in chronic sleep disturbances (highlighting a role of sleep disturbance *per se*), the effects of acute sleep disruption on neurogenesis remain unclear. This may be primarily due to differences in methodologies (e.g., duration of acute sleep disruption, strains, age), but sleep disturbances *per se* may directly affect neurogenesis levels. Another confounding effect could arise from different assessments of neurogenesis and its dynamics. For instance, Junek et al. (2010) combined BrdU (incorporated in the S-phase of the cell cycle only) with two intrinsic cell proliferation markers [proliferating cell neuronal antigen (PCNA) and Ki67 (identifying cells in all phases of the cell cycle)] and showed that although the number of BrdU-labeled cells increased after an acute 12 h sleep deprivation, there was no difference in PCNA- and Ki67-labeled cells compared to controls. This suggested that the apparent rise in cell proliferation could be due to a cell cycle acceleration and not an actual rise in the number of proliferating cells.

It is worth to note that preclinical studies investigating the contribution of sleep to adult hippocampal neurogenesis have been primarily using males. Hence, it would be important to evaluate putative sex differences, especially in the context of neurogenesis-dependent behaviors and the close association of neurogenesis with mood and psychiatric disorders.

## Effect of sleep on adult hippocampal neurogenesis-dependent behaviors

In the previous sections, it has been established that chronic sleep disruption reduces basal levels of neurogenesis in the adult hippocampus. The hippocampus is part of the limbic system and has reciprocal connections with the amygdala and prefrontal cortex, and is thus important in regulating cognitive functions and mood (Pitkanen et al., 2000). It is interesting to consider not only the potential impact of sleep on basal rates of neurogenesis but also on the mediation of changes in neurogenesis associated with hippocampal plasticity, cognition and mood.

The function of adult newborn DG neurons has been involved in many aspects of learning and memory consolidation, such as contextual fear conditioning and long-term spatial memory retention, but discrepancies remain. Importantly, these discrepancies might arise from different environmental regulation and thereby different experiences during the maturation of newborn neurons which play a key role in their integration in hippocampal neural structure (for review Gonçalves et al., 2016).

### Hippocampal-dependent cognitive processes and synaptic plasticity

Sleep has long been thought to play a key role in learning and memory, partly due to the re-activation of neural networks involved in information acquisition, which facilitates consolidation and integration (Walker, 2009; Diekelmann and Born, 2010). A large body of literature describes in humans and animals the importance of sleep before learning and for the consolidation of hippocampal-dependent memory (reviewed in Abel et al., 2013; Vorster and Born, 2015; Krause et al., 2017).

The time of learning and the timing of sleep after learning was shown to be important for performance in hippocampal-dependent episodic-like memory and contextual fear memory in rodents and humans (Palchykova et al., 2006, 2009; Vorster and Born, 2015). Sleep deprivation prior learning also affects the encoding of new hippocampal-dependent memories in rodents and humans (Yoo et al., 2007; Hagewoud et al., 2010). Such effects have been reported using other hippocampal-dependent tasks, such as fear conditioning and episodic memory, but not for non-hippocampal-dependent memories (McDermott et al., 2003; Yoo et al., 2007; Tiba et al., 2008; Van Der Werf et al., 2009). Another study further demonstrated that sleep continuity, with a minimal unit of uninterrupted sleep, was crucial for memory consolidation (Rolls et al., 2011). The authors suggested that this effect was associated with the replay-related events taking place during sleep. A selective role of NREM sleep and REM sleep has also been extensively investigated (Diekelmann and Born, 2010). A recent study demonstrated a causal role of REM sleep theta oscillations by showing that specific inhibition of hippocampal theta oscillations during REM sleep disrupts memory consolidation (Boyce et al., 2016).

The role of sleep for molecular and cellular aspects of hippocampal synaptic plasticity and consolidation has also been extensively studied (for review, e.g., Abel et al., 2013; Kreutzmann et al., 2015; Havekes and Abel, 2017). For instance, recent studies identified some of the molecular and structural mechanisms underlying the detrimental effects of acute short sleep deprivation on memory. These include the attenuation of mTORC1-dependent protein synthesis, reduction of dendritic spine density mediated by the increase activity of the actin-binding protein cofilin, known to play a crucial function in synaptic structure, and the spatial cAMP degradation (Havekes et al., 2016; Tudor et al., 2016).

Overall, the detrimental effects of sleep disruption on neurogenesis, described above, are likely to contribute to the vulnerability of hippocampal networks associated with cognitive impairments.

### Mood, emotion regulation, and depressive disorder

The hippocampus is a limbic structure and contributes to emotional processing and mood disorders, while alterations in adult neurogenesis have been reported in major depression (Miller and Hen, 2015). A growing body of evidence suggests that sleep modulates the regulation of emotions, and sleep disruption has a pervasive effect and is particularly associated with negative emotional experience (Walker, 2009; Harvey, 2011; Palmer and Alfano, 2017).

It is well accepted that sleep disturbances are comorbid with many neurological and psychiatric disorders. This association has been studied for mood disorders, and primarily major depression. Most individuals suffering from depression (i.e., up to 90%) complain about sleep disturbances (Nutt et al., 2008) with a large number experiencing insomnia. Moreover, sleep disturbances are a risk factor to depression, relapse and suicides (Hamilton, 1989; Perlis et al., 1997), and a meta-analysis found that sleep disturbances represent an important risk factor for depression in the elderly (Cole and Dendukuri, 2003). Recent meta-analyses confirmed the occurrence of sleep disturbances by polysomnography recordings in depressed patients, pointing to REM sleep alterations and disturbed sleep continuity (Pillai et al., 2011; Baglioni et al., 2016). Interestingly, REM sleep has been shown to be suppressed by most antidepressant approaches, including antidepressants, electroconvulsive therapy or sleep deprivation (Riemann et al., 2001).

These results suggest that sleep disturbances may mediate the development of depression, and hypnotics (i.e., sleep-promoting drugs) may have beneficial effects. A double-blind placebo-controlled trial reported that co-administration of an antidepressant (fluoxetine; selective serotonin reuptake inhibitor, SSRI) and a hypnotic (eszopiclone; non-benzodiazepine compound acting at GABA_A_ receptors) induced greater benefit in both sleep (i.e., reduced sleep latency and wake after sleep onset and increased total sleep time) and depression assessed by the improved Physician-assessed Clinical Global Impression and HAM-D17 scores, than fluoxetine alone. These beneficial effects were maintained 2 weeks after eszopiclone discontinuation (Krystal et al., 2007), while improvement was no longer observed after 3 months of co-administration of fluoxetine and clonazepam, a benzodiazepine drug (Smith et al., 2002). Zolpidem, the most commonly used hypnotic, also improved sleep in patients with major depressive disorder and persistent insomnia while under antidepressant treatment. Subjective sleep, daytime functioning and well-being were improved by zolpidem (Asnis et al., 1999). In addition, non-pharmacological treatments of insomnia, such as cognitive behavioral therapy, improved the depression and insomnia outcomes (i.e., remission), when provided to patients suffering of insomnia comorbid with depression, with escitalopram as antidepressant treatment (Manber et al., 2008).

These studies suggest that sleep disturbances are a core symptoms of major depression and may have a role in the development of depression. Addressing sleep disruption in mood disorders would improve the treatment of depression.

## Bridging the gap: effects of sleep on cognition and mood through adult hippocampal neurogenesis

**Figure 1:**
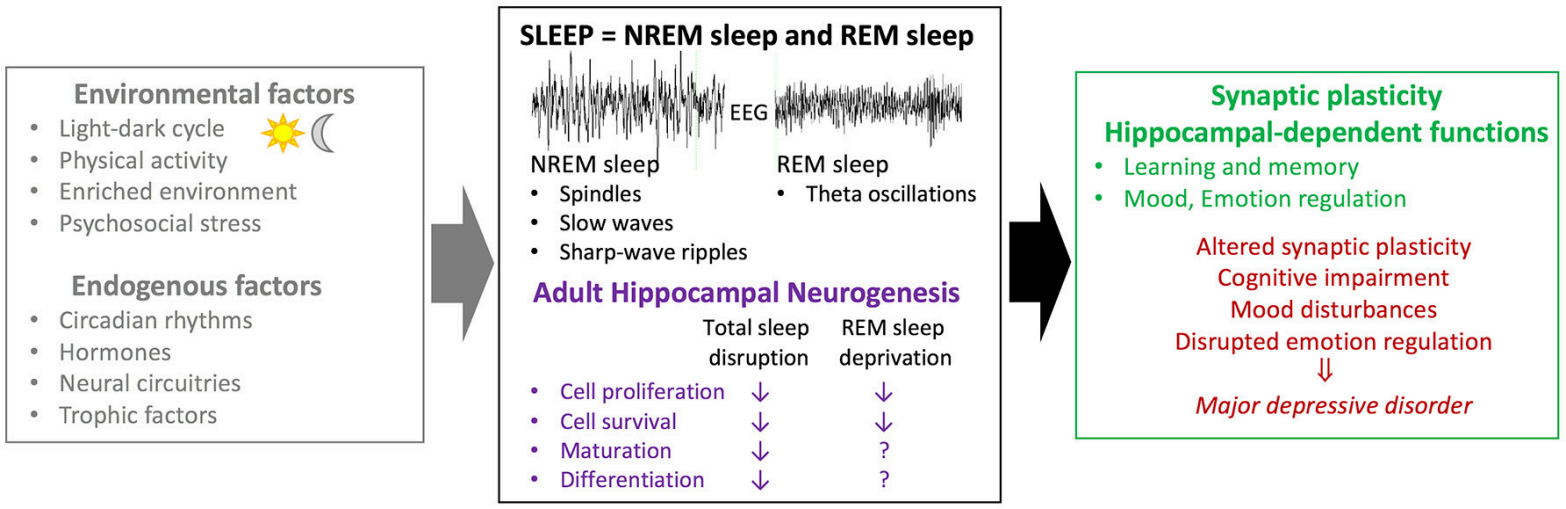
Association between sleep and adult hippocampal neurogenesis in physiological conditions and mood disorders. Several factors including environmental factors (e.g., exercise, physical/psychosocial stress) and endogenous factors (e.g., trophic factors) have been suggested to modulate adult hippocampal neurogenesis. While studies investigating the effects of circadian rhythms are sparse, several lines of evidence suggest that sleep acts as a modulator of adult hippocampal neurogenesis. Sleep disruption (i.e., sleep deprivation; fragmentation; selective REM sleep deprivation) leads to decreased basal rates of cell proliferation and survival in rodents. A selective role for REM sleep on cell maturation and differentiation remain to be clarified. Moreover, sleep disruption has a negative impact on synaptic plasticity and hippocampal-dependant functions. Both sleep disturbances and alterations in adult hippocampal neurogenesis have been associated with major depressive disorders. Based on these different lines of evidence, it can be postulated that sleep effects on hippocampal-dependent functions could be mediated, in part, by altered hippocampal neurogenesis. EEG, electroencephalogram; NREM sleep, non rapid-eye-movement sleep; REM sleep, rapid-eye-movement sleep.

As discussed in previous sections, both neurogenesis and hippocampal-dependent functions are sensitive to sleep. Thus, we hypothesize that the detrimental effects of sleep on cognition and mood might be mediated by a decrease in neurogenesis (Figure 1).

### Cognition–learning and memory

Several studies have reported that learning a hippocampal-dependent task leads to an increase in neurogenesis, while learning deficits are often associated with a decrease in neurogenesis (Gould et al., 1999; Shors et al., 2001; Dobrossy et al., 2003). Blocking neurogenesis also results in decreasing performance in some hippocampal-dependent tasks, such as formation of trace memories (Shors et al., 2002), trace fear conditioning (Madsen et al., 2003), place recognition (Raber et al., 2004), spatial learning in the Morris water maze (Dupret et al., 2007), and pattern separation (Clelland et al., 2009). However, Epp and colleagues recently showed that increasing hippocampal neurogenesis weakens existing memories and facilitates the encoding of new, conflicting (but not non-conflicting) information in mice. Conversely, decreasing neurogenesis stabilizes existing memories, and impedes the encoding of new, conflicting information (Epp et al., 2016). It is also worth noting that learning affects newborn neurons differently depending on their relative ages. For instance, survival is enhanced in slightly mature neurons whereas apoptosis is induced in immature cells (Lemaire et al., 2000). These two processes are inter-linked, and blocking one pathway inhibits the other one, while also impairing memory. Subsequently, memory consolidation requires both addition and removal of neurons depending on their age and functional relevance.

Conditions enhancing or reducing neurogenesis are often associated, respectively, with enhanced (Kempermann et al., 1997; van Praag et al., 1999; Leuner et al., 2004; Mirescu and Gould, 2006) or disrupted (Kuhn et al., 1996; Diamond et al., 1999; Eisch et al., 2000; Gross, 2000; Lemaire et al., 2000; Drapeau et al., 2003; Scerri et al., 2006) hippocampal-dependent learning and memory. Since both hippocampal-dependent learning/memory and adult hippocampal neurogenesis are sensitive to sleep disruptions, the negative effects of sleep loss or sleep fragmentation on hippocampal-dependent learning and memory could be partly due to reduced neurogenesis, contributing to the vulnerability of hippocampal circuitries.

One study (Sportiche et al., 2010) investigated the impact of 4-days sleep deprivation on neurogenesis and spatial vs. non-spatial learning using a water maze paradigm in rats. Subjects that had learned the hippocampal-dependent task showed enhanced cell survival, which was abolished following sleep deprivation. Unexpectedly, sleep-deprived rats showed impaired performance in the spatial task, but enhanced performance in the non-spatial task. In another study (Backhaus et al., 2006), rats subjected to sleep fragmentation for 12 days showed a 32% reduction of BrdU labeled cells compared to yoked and home-cage controls. These subjects were also trained in a Barnes maze paradigm, and the progressive decline in escape time observed in control animals was slower in sleep-fragmented rats which also showed more random, non-spatial search strategies. These results reinforce the hypothesis that the detrimental effects of sleep disruption on cognitive performance might be mediated through a decrease in neurogenesis which has also been proposed to be implicated in cognitive flexibility (Gonçalves et al., 2016; Anacker and Hen, 2017).

Theories of memory formation acknowledge the importance of remodeling and alteration of synaptic strength in neuronal networks, but also the role of sleep in architecting these plasticity processes (Schmidt-Hieber et al., 2004; Stickgold, 2005; Stickgold and Walker, 2005). Therefore, the integration, the maturation and the synaptic strengthening of new neurons to an existing neuronal circuitry could be a complementary form of plasticity. Newborn neurons display robust long-term potentiation and show a lower threshold for their induction (Ramirez-Amaya et al., 2006; Curtis et al., 2007) making them strong candidates to be recruited by behavioral activation (Videbech and Ravnkilde, 2004; Kee et al., 2007).

An increasing interest in the role of glia in neurogenesis has recently emerged. For instance, microglia has been shown to regulate the proliferation and differentiation of neural progenitor cells as well as to control the resulting number of newborn neurons by phagocytosis (Sato, 2015). Astrocytes and gliotransmission have also been implicated in the regulation of sleep homeostasis and working memory deficits induced by sleep deprivation via the adenosine A1 receptor (Halassa et al., 2009; Frank, 2013).

### Mood–depression

There is strong evidence regarding the involvement of the hippocampus in depression. fMRI studies have reported reduced hippocampal volume in depressed patients (MacQueen et al., 2003; Campbell et al., 2004), and the magnitude of this volume reduction was correlated with the frequency, as well as the severity of the pathology (Sheline, 2000). Structural changes such as gray matter alterations (Frodl et al., 2002) might be reversed during remission (Bremner and Vermetten, 2004), and then subsequently associated with fluctuating levels of adult neurogenesis. Many depressed patients show hippocampal-dependent cognitive deficits (Brooke et al., 1994) which suggest hippocampal dysfunction (Brooke et al., 1994; Beason-Held et al., 1999). Antidepressants have been reported to act at the level of the hippocampus, such as SSRIs and the multimodal antidepressant vortioxetine (Dale et al., 2016), and to restrain the hippocampal volume reduction (Sheline et al., 2003) while improving cognition (Bremner and Vermetten, 2004).

Post-mortem studies investigating the difference in neurogenesis in depressed *versus* healthy subjects are inconsistent. For instance, a study reported an increase in neurogenesis in patients treated with antidepressants, whereas no difference between healthy and depressed subjects was observed (Boldrini et al., 2009). By contrast, other studies showed either fewer progenitor cells in depressed patients and no difference following antidepressant treatment (Lucassen et al., 2010) or no change at all compared to healthy patients (Reif et al., 2006). These discrepancies may be due to variations in medication dose and duration, age, length of the disorder, and/or to focusing on the proliferation stage of neurogenesis only.

However, it does not seem that changes in neurogenesis are involved in the etiology of depression. Indeed, suppression of neurogenesis alone in rats did not lead to depressive or anxious phenotypes (Santarelli et al., 2003; Wang et al., 2008; Jayatissa et al., 2009; Fuss et al., 2010) while mice did show neither enhanced depressive-like behaviors (Eisch and Petrik, 2012). However, mice showing lower levels of neurogenesis are more susceptible to stress and display depressive-like phenotypes (Egeland et al., 2017). That could support the hypothesis that a decrease in neurogenesis would need to be associated with genetic predisposition or an environmental trigger such as stress in adulthood to lead to depression. For instance, adult neurogenesis has been proposed to play a role in stress resilience (reviewed in Levone et al., 2015; Besnard and Sahay, 2016).

On the other hand, neurogenesis seems to be related to the behavioral symptoms of depression and their treatment. An increase in neurogenesis was observed following several antidepressant administrations in rodents, including SSRIs, monoamine-oxidase inhibitors, serotonin-norepinephrine reuptake inhibitors and tricyclic antidepressants, all of them serotonin enhancers (Malberg et al., 2000; Czeh et al., 2001; Li et al., 2004; Xu et al., 2006), as well as in non-human primates (i.e., fluoxetine) (Perera et al., 2007). Furthermore, the mechanisms of action of these compounds have been shown to be specific to the SGZ (Malberg et al., 2000; Santarelli et al., 2003; Perera et al., 2007). Studies also reported that blocking neurogenesis in mice abolished the positive effect of antidepressant treatment (Santarelli et al., 2003; Surget et al., 2008). A specific role of neurogenesis in mediating antidepressant treatment action would also explain the delayed onset of therapeutic action. For example, it has been reported that neurogenesis increases following 14- or 28-days of fluoxetine administration, which coincides with the time required for therapeutic action to start, but such effects were not observed following 1- or 5 days of treatment (Malberg et al., 2000). Similar effects regarding neurogenesis modulation have been observed by using psychotropic medications reporting antidepressant effects (Chen et al., 2000; Malberg et al., 2000; Santarelli et al., 2003; Kodama et al., 2004; Nixon and Crews, 2004), as well as non-pharmacological treatments such as electroconvulsive therapy (Scott et al., 2000; Perera et al., 2007) or exercise (van Praag et al., 2005). In addition, predisposing factors to depression, such as chronic stress (Czeh et al., 2001; Coe et al., 2003; Pham et al., 2003), alcohol abuse (Nixon and Crews, 2004), opioid use (Eisch et al., 2000; Krystal, 2012; Scherrer et al., 2016), and hypothyroidism (Madeira et al., 1991) also alter neurogenesis. However, it is worth noting that antidepressants might show both neurogenesis- dependent and -independent effects (Sahay and Hen, 2007; David et al., 2009). For example, in some but not all behavioral paradigms, an inhibition of neurogenesis leads to the effects of fluoxetine being blocked (David et al., 2009), suggesting that other brain regions such as the cingulate cortex could also mediate antidepressant action (Hajek et al., 2008).

As both neurogenesis and depression are linked to sleep, it would be reasonable to assume that sleep could partly affect depression through neurogenesis, and subsequently that the antidepressant-like effects of hypnotics could be linked to increased neurogenesis. Twice daily administration of eszoplicone during 2 weeks increased cell survival by 46% in the SGZ/granule cell layer in rats while having no effect on cell proliferation. Most of these cells showed BrdU-NeuN double-labeling confirming their neuronal identity (Methippara et al., 2010). Yet, another study (Su et al., 2009) reported that chronic co-treatment with eszoplicone and fluoxetine increased cell survival by 50% but again had no effect on cell proliferation; the observed effects were significantly higher when drugs were co-administered compared to single drug treatment. However, acute and chronic administration of zolpidem did not show similar results (Takase et al., 2009). Nevertheless, that could be explained by the fact that newborn neurons do not express GABA_A_ receptor alpha 1 subunit to which binds zolpidem.

How newly born neurons contribute to mood or to symptoms of depression is still not fully understood. The cognitive impairments observed in depression which can be modulated by neurogenesis are also observed in other psychiatric disorders. Decreased hippocampal volumes are reported in patients with schizophrenia, addiction, dementia and anxiety (Kempermann et al., 2008; Thompson et al., 2008; Revest et al., 2009), suggesting that neurogenesis might be an important mediating factor in the pathogenesis. A relevant neuronal mechanism could thus be a decrease in neurogenesis altering the mean age and overall characteristics of neurons in the dentate gyrus, subsequently influencing hippocampal networks' properties and function (Lucassen et al., 2010). Although new adult-born granule cells are in minority amongst cells generated during development, they have been reported to induce an overall increase in dentate gyrus activity and to have a disproportionate influence on hippocampal circuitry and behavior. The way these scarce newborn neurons have an impact on global brain function could be due to their capability to serve both as encoding units and modulators of the patterns and timings of more mature neurons (Ming and Song, 2011).

## Discussion

There is accumulating evidence supporting a role for sleep as a mediator of neurogenesis and its effects on cognition and mood. However, there are certain limitations which must be acknowledged. Regarding cognition, different hippocampal-dependent memories are sensitive to different sleep stages, not necessarily REM sleep primarily associated with a decrease in neurogenesis. In addition, many animal and human studies investigating the impact of sleep on cognition only disturb sleep acutely whereas chronic sleep disruptions have been consistently associated with decreased neurogenesis. This could come from the fact that learning-associated neurogenesis is more sensitive than basal neurogenesis. With respect to mood, contradictory results have been obtained when using different hypnotics or looking at post-mortem studies while the variability in subjects as well as in methodologies makes comparisons difficult. It is worth noting there is also some evidences of an improvement of depressive symptoms after acute sleep deprivation (Giedke and Schwarzler, 2002) but this does not rule out a possible opposing effect of chronic sleep disruption on both depression and neurogenesis. Furthermore, patients suffering from mood disorders could be “reset” following a one night sleep deprivation procedure, questioning again the complex relationship of sleep disturbances with neurogenesis (Krystal, 2012).

The “neurogenic interactome” (Eisch and Petrik, 2012) aims to explain some of these discrepancies by considering diverse neurogenesis altering factors, reciprocal connections of the hippocampus and other areas and behavioral consequences. This makes the link between the effect of changes in neurogenesis on cognition with those observed on mood. Decreased neurogenesis may be detrimental for pattern separation (i.e., the differentiation of similar memories with overlapping hippocampal inputs into different representations; McClelland et al., 1995) and thus lead not only to impaired learning and memory, but also to the inability to adequately discern danger or stress signals. Mueller and colleagues (Mueller et al., 2015) also alluded to pattern separation, suggesting de-correlation of entorhinal cortex inputs leading to formation of separate memory representations is involved in antidepressant action. Kempermann and colleagues (Kempermann, 2008) proposed adult hippocampal neurogenesis enables adaptation of this network to novelty and complexity encountered throughout life which would improve its functioning by separating units of information. The emotional role of the hippocampus would be a consequence of memories being linked to emotional information.

The relationship between neurogenesis and sleep is still a relatively recent research field and it will greatly benefit from further research. Studies comparing mice and rats, as well as different strains and sex, could clarify discrepancies. A key experiment would involve subjecting rodents to sleep disruption while selectively pharmacologically preserving hippocampal neurogenesis to observe whether the cognitive and behavioral deficits are still observed. It would also be of interest to investigate the following: whether the reduction in neurogenesis induced by sleep disruption is normalized by antidepressants, the differential effects of neurogenesis in the ventral and dorsal dentate gyrus, the underlying biochemical and molecular mechanisms of sleep disturbances on neurogenesis, and the effects of disrupted neurogenesis in structures downstream of the hippocampus. Technological advances (e.g., pharmacogenetic ablation of neurogenesis) could potentially allow for the reversible block of neurogenesis in rodents to investigate its relationship with sleep in specific stages of learning and memory or specific timings in mood disorders. It would also be useful to establish standardized labeling paradigms as the effects on younger and older newborn neurons may be different.

This may lead to a better understanding of the role of sleep and neurogenesis in the adult hippocampus and the potential clinical benefits in preventing and treating cognitive disorders and psychiatric diseases.

## Author contributors

CN carried out the literature review research and wrote the article. OB reviewed and revised the literature and the article. RW revised the literature review and contributed to drafting the manuscript. ST conceived, supervised and revised the literature review.

### Conflict of interest statement

The authors declare that the research was conducted in the absence of any commercial or financial relationships that could be construed as a potential conflict of interest.
